# *TaSnRK2.9*, a Sucrose Non-fermenting 1-Related Protein Kinase Gene, Positively Regulates Plant Response to Drought and Salt Stress in Transgenic Tobacco

**DOI:** 10.3389/fpls.2018.02003

**Published:** 2019-01-14

**Authors:** Jialu Feng, Lianzhe Wang, Yanan Wu, Qingchen Luo, Yang Zhang, Ding Qiu, Jiapeng Han, Peipei Su, Zhiyong Xiong, Junli Chang, Guangxiao Yang, Guangyuan He

**Affiliations:** ^1^The Genetic Engineering International Cooperation Base of Chinese Ministry of Science and Technology, Key Laboratory of Molecular Biophysics of Chinese Ministry of Education, College of Life Science and Technology, Huazhong University of Science and Technology, Wuhan, China; ^2^School of Life Sciences and Engineering, Henan University of Urban Construction, Pingdingshan, China; ^3^Key Laboratory of Herbage and Endemic Crop Biotechnology, Ministry of Education, College of Life Sciences, Inner Mongolia University, Hohhot, China

**Keywords:** wheat, *TaSnRK2.9*, abiotic stress, antioxidant, ROS, ABA, SnRK-ABF

## Abstract

Sucrose non-fermenting 1-related protein kinase 2 (SnRK2) family members play crucial roles in plant abiotic stress response. However, the precise mechanism underlying the function of SnRKs has not been thoroughly elucidated in plants. In this research, a novel *SnRK2* gene, *TaSnRK2.9* was cloned and characterized from common wheat. The expression of *TaSnRK2.9* was upregulated by polyethylene glycol (PEG), NaCl, H_2_O_2_, abscisic acid (ABA), methyl jasmonate (MeJA), and ethrel treatments. *TaSnRK2.9* was mainly expressed in wheat young root, stamen, pistil, and lemma. Overexpressing *TaSnRK2.9* in transgenic tobacco enhanced plants’ tolerance to drought and salt stresses both in young seedlings and mature plants with improved survival rate, seed germination rate, and root length. Physiological analyses suggest that TaSnRK2.9 improved antioxidant system such as superoxide dismutase (SOD), catalase (CAT), peroxidase (POD), and glutathione (GSH) to reduce the H_2_O_2_ content under drought or salt stress. Additionally, *TaSnRK2.9* overexpression plants had elevated ABA content, implying that the function of TaSnRK2.9 may be ABA-dependent. Moreover, TaSnRK2.9 increased the expression of some ROS-related, ABA-related, and stress-response genes under osmotic or salt treatment. TaSnRK2.9 could interact with NtABF2 in yeast two-hybrid assay, and increased the expression of *NtABF2* under mannitol or NaCl treatment in transgenic tobacco plants. In conclusion, overexpression of *TaSnRK2.9* in tobacco conferred plants tolerance to drought and salt stresses through enhanced ROS scavenging ability, ABA-dependent signal transduction, and specific SnRK-ABF interaction.

## Introduction

Abiotic stresses such as drought and high salinity severely affect plants growth, development, and crop productivity (Ruizlozano et al., 2012). To cope with such abiotic stresses, plants have evolved a series of complex physiological mechanisms such as ROS detoxification, ABA signaling pathway, ion balance, and osmosis regulation (Zhang et al., 2018). Previous researches showed that many genes, such as transcription factors and protein kinase genes, participated in response to abiotic stresses. And the SnRK2 plays a key role in plant stress signal transduction pathway.

The CDPK-SnRK superfamily includes seven families of serine-threonine protein kinases: CDPKs, CRKs, PPCKs, PEPRKs, CaMKs, CCaMKs, and SnRKs (Hrabak et al., 2003). In eukaryotic cells, yeast sucrose non-fermenting 1 (SNF1) protein kinase, animal AMPK, and plant SnRK protein kinase are highly conserved and take an active part in plants growth and development processes. Plant SnRKs are divided into three subgroups (SnRK1, SnRK2, and SnRK3) based on their structure difference (Hardie et al., 1998). SnRK1 mainly regulates carbon and nitrogen balance, while SnRK2 and SnRK3, which are plant-specific, play an essential role in stress signal transduction (Hrabak et al., 2003).

Increasing evidences suggest that PYR/PYL/RCARs-type 2C phosphatase (PP2C)-SnRK2 is an ABA-dependent stress signal pathway which is important in plant stress response (Rosenberger and Chen, 2018; Zhao et al., 2018). ABA is an indispensable phytohormone that plays a crucial role in plant stress response (Soma et al., 2017; Vishwakarma et al., 2017; Wang P. et al., 2018). Under normal condition, PP2C inhibited SnRK2s, while subjecting to abiotic stresses, accumulated ABA can be sensed by PYR/PYL/RCAR proteins, resulting in PP2Cs inhibition. SnRK2s are then activated by self-phosphorylation, which in turn induces the expression of a series of ABA-regulated genes (Melcher et al., 2009; Nishimura et al., 2009; Park et al., 2009; Umezawa et al., 2009; Vlad et al., 2009). Many researches show that SnRK2s are positively regulated by ABA and play key roles in plant abiotic stress (Umezawa et al., 2013).

In *Arabidopsis*, *SnRK2s* (*AtSnRK2.2*, *AtSnRK2.3*, *AtSnRK2.6*, *AtSnRK2.7*, and *AtSnRK2.8*) function redundantly in response to ABA and abiotic stresses (Fujii and Zhu, 2009; Fujita et al., 2009; Nakashima et al., 2009; Mizoguchi et al., 2010). In poplar, *PtSnRK2.5* and *PtSnRK2.7* positively regulate the plants response to salt stress (Song et al., 2016). In wheat, ten *SnRK2s* were identified by bioinformatic analyses (Zhang et al., 2016). Overexpressing *TaSnRK2.3* or *TaSnRK2.4* in *Arabidopsis* conferred plants enhanced tolerance to drought, salt, and freezing stresses through improved expression of stress-response genes and physiological indices such as decreased water loss and increased osmotic potential (Mao et al., 2010; Tian et al., 2013). Overexpressing *TaSnRK2.7* or *TaSnRK2.8* in *Arabidopsis* conferred plants tolerance to drought, salt, and cold stresses with longer primary roots and various physiological characteristics including strengthened cell membrane stability and improved photochemical efficiency (Zhang et al., 2010, 2011). The four identified genes all located in the cell membrane, cytoplasm, and nucleus. They all responded to PEG and NaCl. *TaSnRK2.3, TaSnRK2.7*, and *TaSnRK2.8* responded to cold but *TaSnRK2.4* did not. *TaSnRK2.7* did not respond to ABA, suggesting that TaSnRK2.7 might be involved in non-ABA-dependent signal transduction pathways.

In this study, *TaSnRK2.9* was cloned and functionally characterized from common wheat. To investigate the precise role of *TaSnRK2.9* in plant stress response, *TaSnRK2.9* was ectopically expressed in tobacco. Our results showed that *TaSnRK2.9* overexpression plants exhibited enhanced tolerance to drought and high salinity in young seedlings and mature plants. Further investigation suggests that *TaSnRK2.9* improved plant stress tolerance through detoxification of ROS, regulating the expression of ABA-related genes, and interacting with ABFs.

## Materials and Methods

### Wheat Growth Conditions and Stress Treatments

Common wheat (*Triticum aestivum* L., cv. Chinese spring) was used in this study. Wheat seeds were surface-sterilized with 75% (v/v) ethanol for 2 min followed by washing three times in double distilled water. Sterilized seeds were germinated and cultured with water in a climate chamber under a 12 h light/12 h dark cycle at 22°C with light intensity of 150 μmol m^-2^s^-1^. Two-week-old wheat seedlings with two leaves, were treated by multiple abiotic stresses or signaling molecules. Different stress treatments were carried out by submerging wheat seedling roots in solutions of 20% PEG6000, 200 mM NaCl, and 10 mM H_2_O_2_, respectively. For treatments with signaling molecules, seedlings were sprayed with 100 μM ABA, 100 μM MeJA, and 100 μM ethrel, respectively. Wheat seedlings cultivated in water were used as control. Leaves and roots of the seedlings were sampled at different time points (0, 1, 3, 6, 12, and 24 h) after treatments. Wheat plants grown in the field were used to determine organ specific expression of *TaSnRK2.9*. Young root, young stem, young leaf (at the four-leaf stage), flag leaf, mature stem, mature leaf (at anthesis stage), stamen, pistil, lemma, coleoptile, young spike, and mature spike were sampled. All obtained samples were immediately frozen in liquid nitrogen and stored at -80°C for RNA extraction and subsequent qRT-PCR analysis.

### Cloning and Identification of *TaSnRK2.9* Gene

To identify *TaSnRKs* in wheat, *in silico* cloning was used to predict putative *TaSnRK* genes. The *Triticum urartu* gene *TuSAPK9* (GenBank: EMS68031.1) with high homology to *OsSAPK9* was used as a query probe to blast the EST library of wheat. Then we obtained a wheat EST (HX198182) belonging to *TaSnRK* family. Since the EST did not show a clear 3′-end, SMART RACE cDNA amplification kit (Clontech, United States) was used to amplify the 3′ end of the gene. Then the full-length cDNA sequence was obtained. The PCR products were cloned into a pMD18-T vector (TaKaRa, Dalian, China) and transfected into *E. coli* TOP 10 competent cells (Tiangen, Beijing, China). Finally, the target gene in positive clone was sequenced (TSINGKE BioTech, Beijing, China) and analyzed by BLAST.

### Bioinformatics Analysis of *TaSnRK2.9*

Protein sequences analysis was conducted with Clustal W. Then GeneDoc software was used to edit the alignment results. MEGA was selected to construct the phylogenetic tree using the neighbor-joining method (He et al., 2017). All the sequences were downloaded from NCBI database^1^.

### qRT-PCR

Total RNA was extracted from different samples collected above according to Plant Tissue Total RNA Extraction kit (Zomanbio, Beijing, China). Three microliters RNA was used in agarose gel electrophoresis to check the quality and integrity of the obtained RNA samples. First strand cDNA was synthesized from mRNA using RevertAid First Strand cDNA Synthesis Kit (Thermo Fisher Scientific, Lithuania). Expression patterns of *TaSnRK2.9* in different wheat organs and under different stress treatments, signal molecules were analyzed by qRT-PCR using the SuperReal PreMix Plus (SYBR Green) (Tiangen, Beijing, China) on a Fluorescence Real-time PCR detection system (Bio-Rad Laboratories, Hercules, CA, United States). PCR was conducted to test efficiency and specificity of the primer pairs, the amplified product was sequenced. *TaActin* (GenBank: AB181991.1) was used as the internal reference gene. Each sample was measured using three biological replicates. Appropriate negative controls without template were used to check any possible contamination or primer dimers in every single experiment. The relative expression of mRNA was calculated using the 2^-ΔΔCt^ formula.

### Subcellular Localization of TaSnRK2.9 Protein

To determine the subcellular localization of TaSnRK2.9 protein, a recombinant construct of pCAMBIA1304::*TaSnRK2.9::GFP* was transformed into onion epidermal cells using a gene gun (PDS-1000; BIO-RAD, Hercules, CA, United States) in accordance with the instruction manual. Restriction sites were added to the 5′ and 3′ ends of the complete coding sequence without the termination codon of *TaSnRK2.9* by PCR using specific primer pairs. The product was subsequently inserted into pCAMBIA1304 vector to generate the TaSnRK2.9-GFP fusion protein under control of the *CaMV 35S* promoter. Then the pCAMBIA1304-TaSnRK2.9-GFP fusion protein construct was introduced into the onion epidermal cells with a gene gun. The pCAMBIA1304-GFP vector was used as a control. After incubation at 20°C for 24 h in the dark, the tissues were examined by fluorescence microscopy (IX71, Olympus, Japan).

### Yeast Two-Hybrid Assay

Yeast two-hybrid assay was used to investigate the interaction between TaSnRK2.9 and NtABFs *in vivo*. The complete coding sequences of *TaSnRK2.9*, *NtABF1*, *NtABF2*, and *NtABF4* were amplified by PCR using primers containing specific restriction sites. *TaSnRK2.9* PCR products were inserted into pGADT7 vector. While *NtABF1*, *NtABF2*, and *NtABF4* PCR products were cloned into pGBKT7 vector to create pGADT7-*TaSnRK2.9*, pGBKT7-*NtABF1*, pGBKT7-*NtABF2*, and pGBKT7-*NtABF4* constructs. These recombinant constructs were transformed into yeast strain AH109 by the lithium acetate method. Plasmid pGADT7-T and pGBKT7-lam were used as a negative control, while pGADT7-T and pGBKT7-53 were used as a positive control. After confirmation by screening on selective medium plates (SD/-Trp-Leu), the positive colonies were transferred onto SD/-Trp-Leu-His-Ade plates with X-α-D-Galactosidase (X-α-gal). The protein interaction was determined according to the growth status on the plates after 3 days incubating under 30°C in the dark.

### Bimolecular Fluorescence Complementation Assay

For the bimolecular fluorescence complementation (BiFC) assay, recombinant constructs TaSnRK2.9-YNE and NtABF2-YCE were constructed and transferred into *Agrobacterium tumefaciens* strain EHA105, respectively. Then they were co-transformed into tobacco leaves using transient expression method with a medical syringe. The tobacco plants were placed under normal growth condition after injection. Fluorescence was observed by fluorescence microscope (IX71, Olympus, Japan) 36 h after transformation.

### Plant Transformation and Establishment of Transgenic Plants

To generate transgenic plants, the recombinant plasmids pCAMBIA1304-*TaSnRK2.9* under the control of the *CaMV 35S* promoter was introduced into *A. tumefaciens* strain LBA4404. According to an *Agrobacterium*-mediated leaf disk transformation method, the pCAMBIA1304-*TaSnRK2.9* and empty vector pCAMBIA1304 were transformed into tobacco leaves, respectively. Specific primers were used to confirm positive seedlings to amplify *TaSnRK2.9* by PCR. Then positive seedlings were transferred to soil for further study. T_1_ seeds were germinated and grown on MS medium containing 100 mg/L kanamycin for 2 weeks, then the survived plants were transferred to soil to get T_2_ seeds. Depending on the transgene expression level, three independent transgenic T_3_ line seedlings (OE2, OE3, and OE5) were selected to conduct stress tolerance and all the following experiments.

### Stress Tolerance Analysis of Transgenic Lines

Wild type (WT), VC, and *TaSnRK2.9* overexpression (OE) lines were cultured in MS medium for 1 week and then transplanted into soil. For drought stress tolerance assay, 3-week-old seedlings similar in growth status were subjected to withhold water for 3 weeks. The control plants were watered regularly. Fifty plants from each line were used to detect survival rate after withholding water for 3 weeks. At the 2 week drought stress treatment stage, leaves from each line were collected to measure H_2_O_2_, SOD, CAT, POD, IL, RWC, MDA, proline, soluble sugar, and ABA contents. Three weeks after withholding water, photographs were taken. Then the plants were watered for 1 week for recovery.

For the salt stress treatment assay, the plants were irrigated with 300 mM NaCl solution three times a week for 4 weeks. Meanwhile, SOD, CAT, and POD activities, H_2_O_2_, GSH, MDA, proline, soluble sugar, and chlorophyll contents, and IL were determined after 2 weeks of salt treatment. The abiotic tolerance experiments were repeated three times.

### Measurement of H_2_O_2_ Accumulation, Antioxidants, IL, RWC, MDA, Proline, Soluble Sugar, ABA, and Chlorophyll Content

The leaves from the same position of seedlings were collected to assay the physiological indices. For the assessment of H_2_O_2_, GSH and proline contents, the enzyme activities of SOD, CAT and POD, detection kits (Jiancheng, China) were used by spectrophotometric method. For the MDA content, thiobarbituric acid method was used according to the method described previously (Heath and Packer, 1968). For the IL, the samples were clipped into small pieces, and then were immersed in test tubes with 10 ml of distilled water at room temperature for 12 h. The initial conductivity (C1) was determined with a conductivity meter (DDBJ-350, Shanghai, China). Subsequently, the samples were boiled for 20 min to release all the ions. After cooling to the room temperature, the conductivity (C2) was determined again. IL was calculated as IL (%) = (C1/C2) × 100% (Hu et al., 2012). For the RWC, leaves were collected and weighed as fresh weight (FW), then they were covered with water for 12 h to measure turgid weight (TW). Subsequently, the leaves were put in the dry oven at 105°C for 15 min, then leaves were dried at 80°C to get dried weight (DW), such that RWC (%) = [(FW-DW)/(TW-DW)] × 100% (Luo et al., 2017). For the soluble sugar content, phenol reaction method was used as described before (Kong et al., 2011). For the ABA content, an ABA ELISA assay kit (Jiancheng, China) was used (Wang Y. et al., 2018). For the chlorophyll content, leaves were grinded into pounder, then soaked in 50 mL ethanol (90%, v/v) tube, and the samples were incubated in a shaker at low temperature for 3 h. The chlorophyll content was analyzed by a spectrophotometer using light at the wave lengths of 665 and 649 nm.

### Expression Analyses of Stress-Related Genes

Two-week-old seedlings grown in the MS medium were transplanted to MS medium with 300 mM mannitol or 200 mM NaCl for 7 days. And the whole seedlings were sampled for RNA extraction after treatments. The qRT-PCR was used to analyze the expression of stress-related genes. Additionally, *NtActin* was used as a reference gene. All primers used in this study are listed in Supplementary Table S1.

### Statistical Analysis

Statistical analyses were performed with *Excel*. All the experiments were carried out at least three times, and the Student’s *t*-test was applied to compare the difference, at a remarkable difference *P* < 0.05 or *P* < 0.01 (Wang X.et al., 2015).

## Results

### Identification of *TaSnRK2.9* Gene From Wheat

A full length cDNA of *SnRK* was cloned from wheat (Chinese spring) using RACE method. Sequence alignment and phylogenetic analysis (Supplementary Figure S1) showed that it had high similarity to *OsSAPK9*. Therefore, it was designated *TaSnRK2.9* (GenBank accession No. MH844552). *TaSnRK2.9* consists a 1080 bp ORF which encodes a 360 amino acid protein with a predicted relative molecular mass of 40.36 kDa and theoretical pI of 4.94. Sequence blast using the Ensembl Plants database^2^ showed that *TaSnRK2.9* is located on wheat 5A chromosome.

### Expression Patterns of *TaSnRK2.9* Under Various Stress Conditions and in Different Organs

**FIGURE 1:**
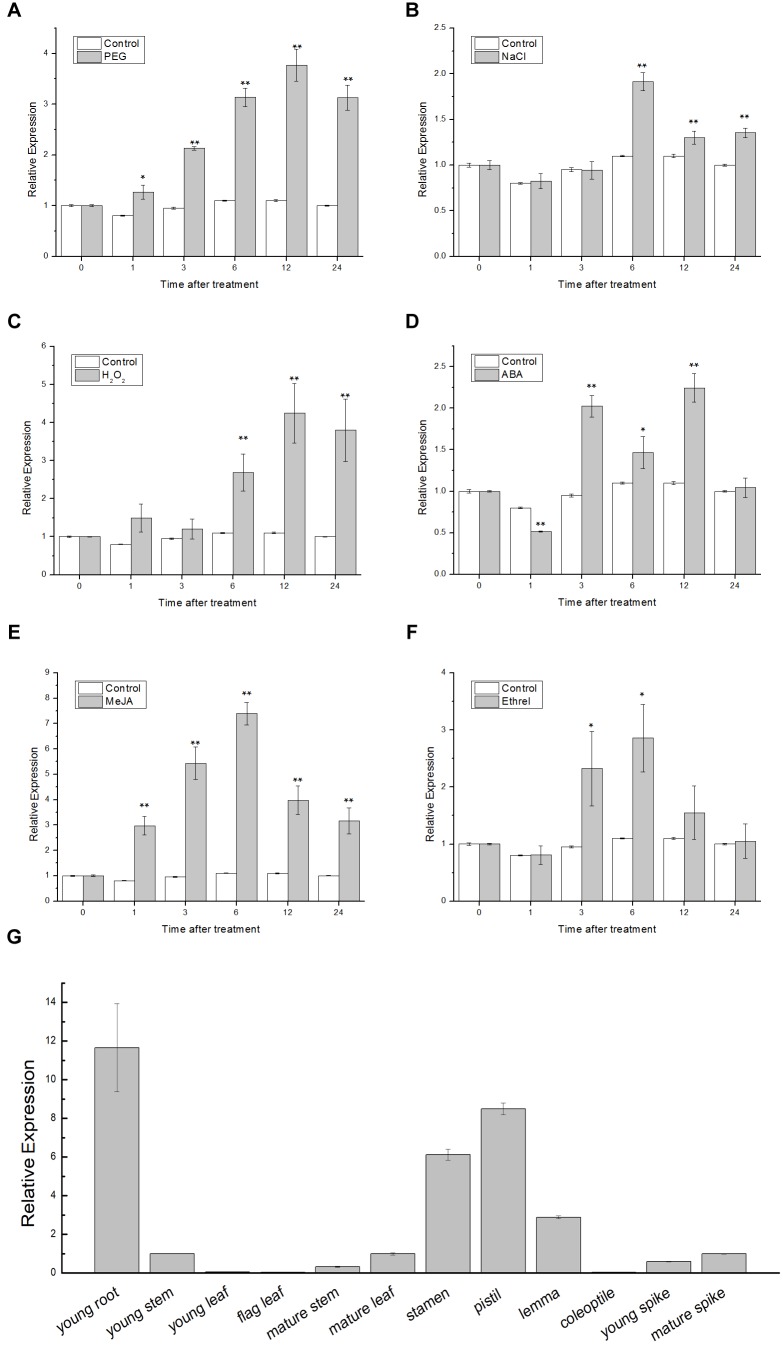
Expression pattern analyses of *TaSnRK2.9* gene in wheat. Two-week-old wheat seedling leaves under different treatments including **(A)** 20% PEG6000, **(B)** 200 mM NaCl, **(C)** 10 mM H_2_O_2_, **(D)** 100 μM ABA, **(E)** 100 μM MeJA, **(F)** 100 μM ethrel. **(G)** Organ-specific expression of *TaSnRK2.9* in young root, young stem, young leaf, flag leaf, mature stem, mature leaf, stamen, pistil, lemma, coleoptile, young spike, and mature spike. qRT-PCR was used to analyze the expression differences. Values are means ± SD from three independent replicates. Asterisks indicate significant difference between WT and the transgenic lines (Student’s *t*-test, ^∗^*P* < 0.05; ^∗∗^*P* < 0.01).

The qRT-PCR was employed to examine the expression patterns of *TaSnRK2.9*. In the stress treatment assay, 1-week-old wheat seedlings were treated with PEG, NaCl, H_2_O_2_, ABA, MeJA, and ethrel, and were sampled at 0, 1, 3, 6, 9, 12, and 24 h, respectively. In organ-specific expression assay, young root, young stem, young leaf, flag leaf, mature stem, mature leaf, stamen, pistil, lemma, coleoptile, young spike, and mature spike from the wheat were collected to extract RNA. As shown in Figures 1A–F, *TaSnRK2.9* was significantly upregulated by PEG, approximately 3.7-fold. *TaSnRK2.9* expression reached the maximum of almost twofold at 6 h after NaCl treatment. With H_2_O_2_, ABA, MeJA, and ethrel treatments, the expression of *TaSnRK2.9* was gradually elevated by 4.2-fold at 12 h, 2.2-fold at 12 h, 7.3-fold at 6 h, and 2.8-fold at 6 h, respectively. These results indicate that the expression of *TaSnRK2.9* is responsive to PEG, NaCl, H_2_O_2_, ABA, MeJA, and ethrel, implying that *TaSnRK2.9* may play a role in abiotic stress response. In addition, *TaSnRK2.9* was expressed in all the organs examined in wheat (Figure 1G). *TaSnRK2.9* expressed at higher levels in young root, stamen, pistil, and lemma, while its expressions were lower in young stem, young leaf, flag leaf, mature stem, mature leaf, coleoptile, young spike, and mature spike.

### TaSnRK2.9 Protein Is Localized Throughout the Cell

**FIGURE 2:**
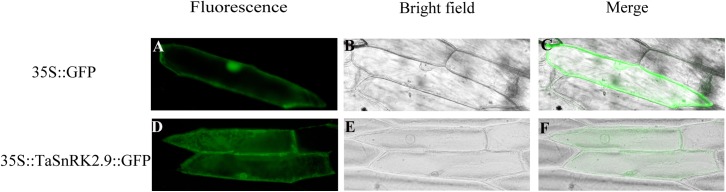
Subcellular localization of TaSnRK2.9 protein. The fusion protein TaSnRK2.9-GFP (pCAMBIA1304-TaSnRK2.9) and GFP (pCAMBIA1304-GFP) were transiently expressed in the onion epidermis cell by the gene-gun method. **(A–C)** Show the onion epidermis cell expressing pCAMBIA1304-GFP as the controls, **(D–F)** show the onion epidermis cell expressing pCAMBIA1304-TaSnRK2.9-GFP fusion protein. Pictures were taken in bright field and fluorescence field by fluorescence microscopy 24 h after bombardment. Three independent biological experiments were performed and produced similar results.

To investigate the subcellular localization of TaSnRK2.9, a recombinant vector 35S::*TaSnRK2.9::GFP* was constructed. Then the recombinant plasmid and the control vector (pCAMBIA1304-GFP) were transformed into onion epidermal cells by a gene-gun method. The green fluorescence signal from the 35S::TaSnRK2.9::GFP was observed in the whole cell, just the same as the control vector 35S::GFP (Figure 2). The results showed that TaSnRK2.9 protein was localized throughout the cell.

### Overexpression of *TaSnRK2.9* Enhances Drought and Salt Tolerance in Mature Transgenic Tobacco Plants

Since the expression of *TaSnRK2.9* is responsive to PEG and NaCl treatments, whether TaSnRK2.9 participates in plant response to osmotic stress was examined. Transgenic tobacco plants overexpressing *TaSnRK2.9* under the control of *CaMV 35S* promoter were generated, and the plants transformed by empty vector severed as vacant (VC). Among these lines, three T_3_ lines, OE2, OE3, and OE5 with higher *TaSnRK2.9* transcript levels were selected for the further investigation.

**FIGURE 3:**
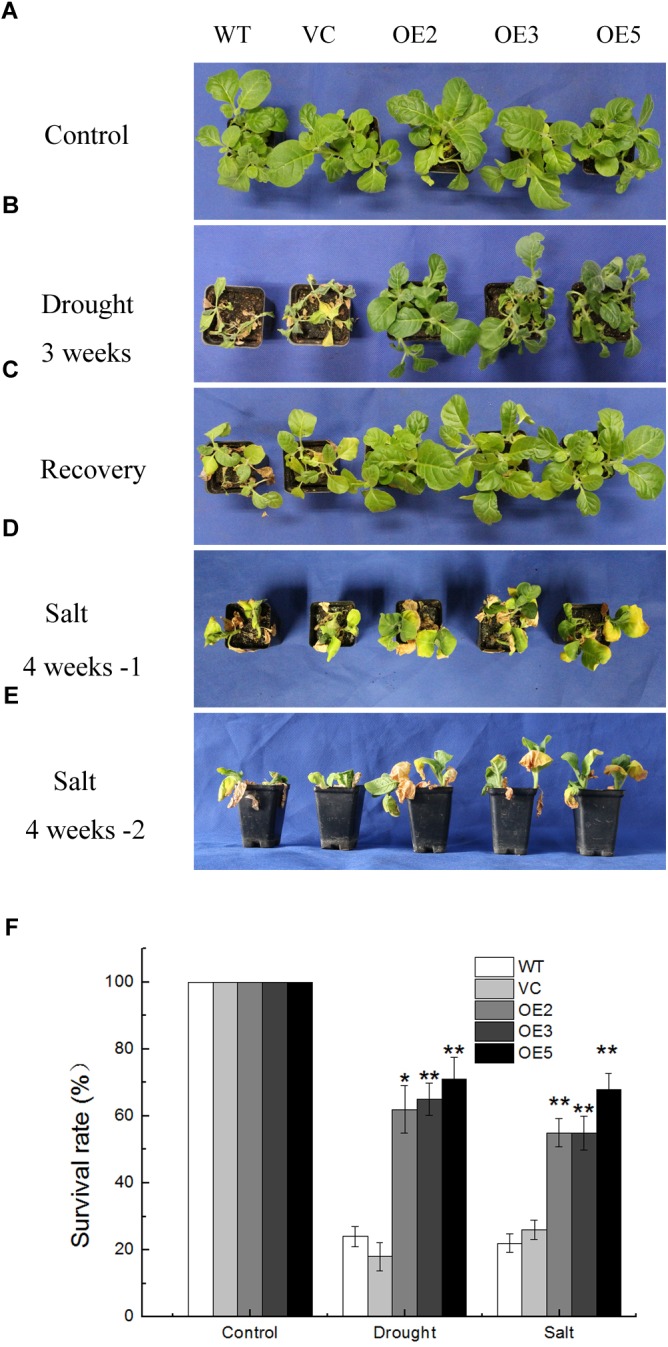
Phenotype and survival rate of the transgenic tobacco plants overexpressing *TaSnRK2.9* under drought and salt stresses. **(A)** Control group of 4-week-old tobacco plants grown in the control environment. **(B)** Tobacco plants after water withholding for 3 weeks. **(C)** Tobacco plants in **(B)** were re-watered for 1 week. **(D,E)** Tobacco plants were subjected to salt condition for 4 weeks, pictures were taken from different orientations. **(F)** The survival rate of *TaSnRK2.9* overexpressing plants and the control under drought or salt conditions. Data in **(F)** are means ± SD from three independent replicates (^∗^*P* < 0.05; ^∗∗^*P* < 0.01 by Student’s *t*-test).

Seeds germinated on MS medium containing 100 mg L^-1^ of kanamycin were transplanted in soil for 2 weeks under normal condition. Then the plants were subjected to drought or salt stress. After withholding water for 3 weeks, the control lines (WT, VC) almost died, the leaves were yellow and shriveled, some were wilted, while the transgenic lines (OE2, OE3, OE5) were still green and grew vigorously with only a few leaves dehydrated (Figure 3B). After re-watering for 1 week, more than 60% of the OE plants survived compared with only 18% of the control plants (Figures 3C,F). For the salt stress, plants were treated with NaCl solution for 4 weeks. After the treatment, transgenic plants grew better than the controls, while leaf wilting was more evident in the control plants (Figures 3D,E). Meanwhile, the survival rates of the *TaSnRK2.9* overexpression tobacco plants (50%) were greatly higher than the control lines (20%) under salt stress (Figure 3F). These results showed that TaSnRK2.9 improved the drought and salt tolerance in mature transgenic tobacco plants.

### Overexpression of *TaSnRK2.9* Increases the Seeds Germination Rate and Root Length at Seedling Stage in Tobacco Under Mannitol or NaCl Conditions

**FIGURE 4:**
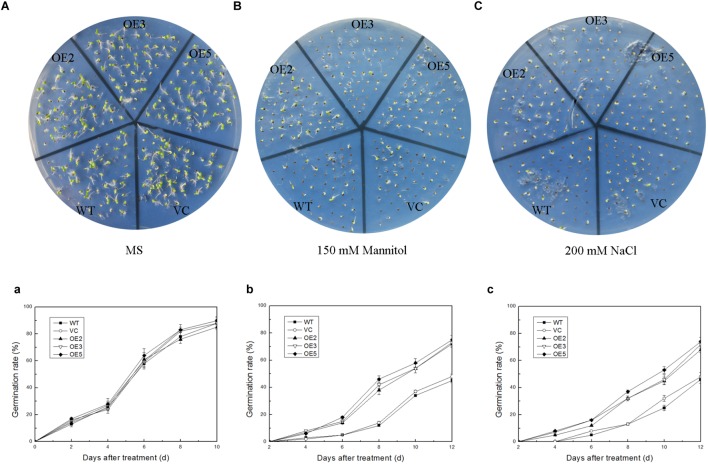
Germination rate of the transgenic tobacco seeds overexpressing *TaSnRK2.9* under osmotic stress. *TaSnRK2.9* overexpressing tobacco and the control (WT and VC) seeds were germinated on MS medium with no mannitol and NaCl **(A,a)**, 150 mM mannitol **(B,b)**, and 200 mM NaCl **(C,c)** and photographs were taken after 2 weeks. Plates **(A–C)** are the photographs of germination status on medium, chart **(a–c)** are calculated data of germination rate. Values are means ± SD (*n* = 3).

**FIGURE 5:**
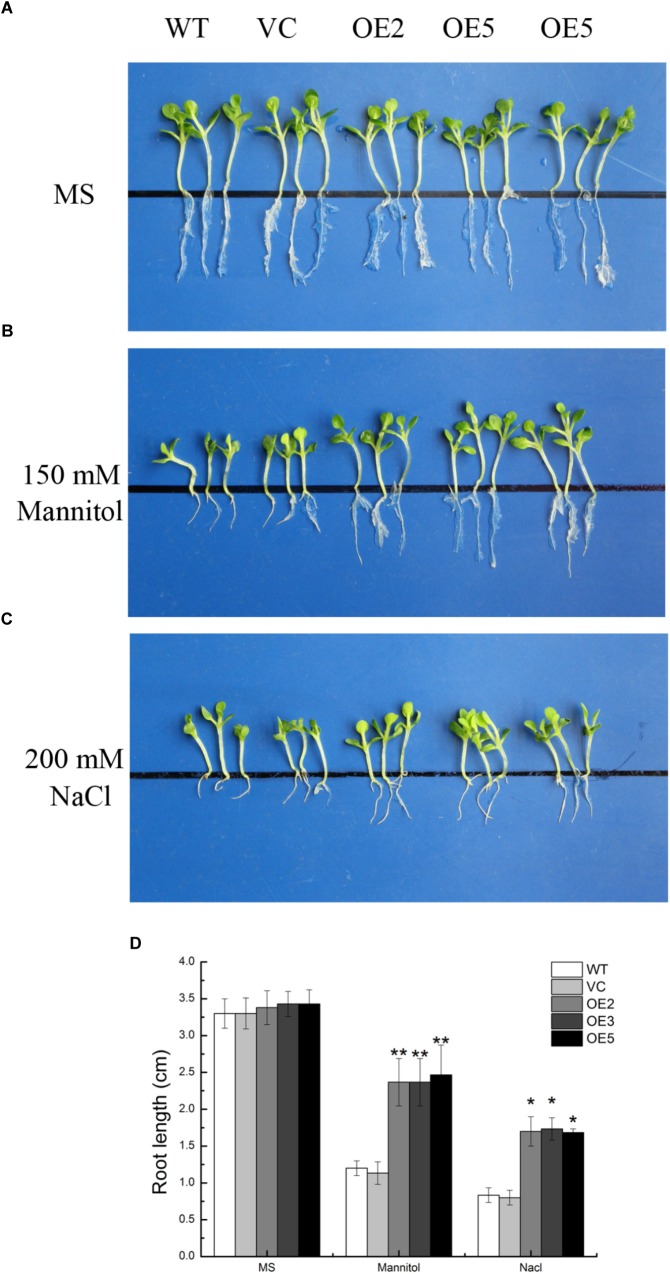
Root elongation of the transgenic tobacco seedlings overexpressing *TaSnRK2.9* under osmotic stress. *TaSnRK2.9* overexpressing tobacco and the control (WT and VC) seedlings were grown on **(A)** MS medium, **(B)** MS medium with 150 mM mannitol, **(C)** 200 mM NaCl for 2 weeks. **(D)** The root lengths were gauged and analyzed. Values are means ± SD (*n* = 3, ^∗^*P* < 0.05; ^∗∗^*P* < 0.01 by Student’s *t*-test).

To investigate the function of TaSnRK2.9 during seed germination and at young seedlings stage, the seeds germination rate and the root elongation were examined. For the germination rate, the seeds were sown on MS medium with 150 mM mannitol, or with 200 mM NaCl. Both control and *TaSnRK2.9* overexpression lines grew similar on the MS medium (Figure 4A,a) under normal condition. When seeds were germinated on MS medium with 150 mM mannitol (Figure 4B,b) or with 200 mM NaCl (Figure 4C,c), the transgenic lines had a higher germination rate than the controls. For the root elongation experiment, the germinated seeds continued to grow on the MS medium until two cotyledons were fully appeared, then the seedlings were transferred to MS medium with mannitol or NaCl. The transgenic seedlings showed longer roots than the controls (WT, VC) under treatments (Figures 5B–D). The results suggested that the TaSnRK2.9 also increase the tolerance of the transgenic tobacco during seed germination and at seedlings stage under osmotic and salt stresses.

### Physiological Indices Comparison Between Transgenic and Control Plants Under Drought or Salt Conditions

That the overexpression of *TaSnRK2.9* enhanced drought and salt tolerance in transgenic seeds, seedlings, and mature plants, prompted us to examine the physiological indices influenced by *TaSnRK2.9* overexpression. Physiological indices were examined after drought or salt treatment. Abiotic stress such as drought and salt could lead to the accumulation of ROS, which results in plants oxidative damage, and ROS scavenging is essential in plant response to abiotic stress (Giraud et al., 2008; Hossain and Dietz, 2016).

**FIGURE 6:**
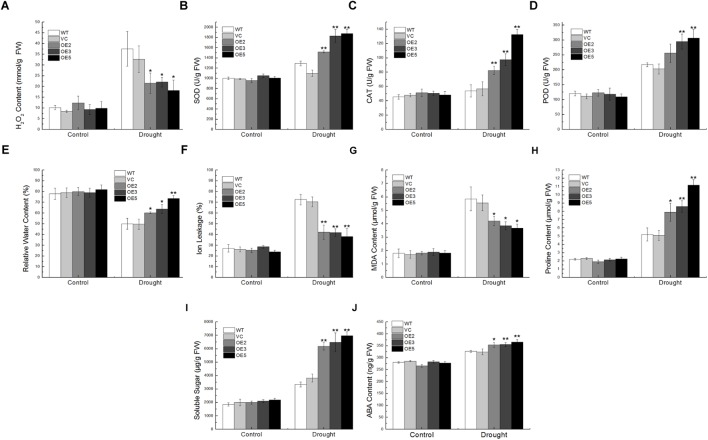
Physiological indices of the transgenic tobacco plants overexpressing *TaSnRK2.9* under drought stress. Two-week-old tobacco plants grown on MS medium were transplanted in soil for 3 weeks. Plants were subjected to drought treatments while the controls were normally watered for 1 week. Leaves of the five lines were sampled for the assessment of **(A)** H_2_O_2_, **(B)** SOD, **(C)** CAT, **(D)** POD, **(E)** RWC, **(F)** IL, **(G)** MDA, **(H)** Proline, **(I)** Soluble sugar, **(J)** ABA. Data are means ± SD (*n* = 3, ^∗^*P* < 0.05; ^∗∗^*P* < 0.01 by Student’s *t*-test).

For the drought treatment, the transgenic lines showed lower H_2_O_2_ content than the controls, and the activity of ROS-scavenging enzymes such as SOD, CAT, POD in the transgenic lines were higher (Figures 6A–D). Transgenic lines exhibited lower IL and more RWC than the WT and VC (Figures 6E,F), indicating that overexpression of *TaSnRK2.9* could keep plants from water lose and retain ions after drought stress. MDA, an indicator of membrane lipid peroxidation, could damage structure and change the permeability of the plasma membrane (Moore and Roberts, 1998). The proline in the plant accumulates under osmotic stress conditions protects plant from adverse environment (Delauney and Verma, 1993). Transgenic lines exhibited lower MDA content than the controls, and the more proline was accumulated in the *TaSnRK2.9* overexpression plants (Figures 6G,H). Meanwhile, the soluble sugar content in the transgenic lines was also higher than the controls (Figure 6I). As the expression of *TaSnRK2.9* could be upregulated by ABA, the ABA content was also examined. The ABA content was increased in the transgenic lines after drought treatment (Figure 6J), which indicates that TaSnRK2.9 may participate in the ABA signal pathway. These results suggested that *TaSnRK2.9* overexpressing plants could enhance the ability of ROS-scavenging, maintain structural integrity of cell membrane, and produce more osmotic substance to meliorate damage imposed by drought stress.

**FIGURE 7:**
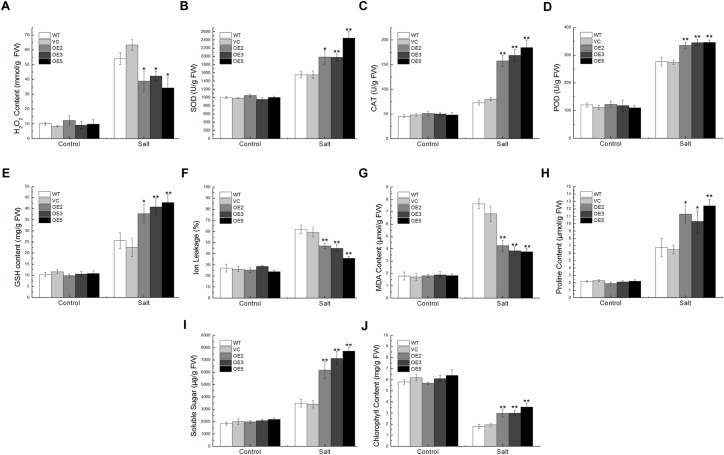
Physiological indices of the transgenic tobacco plants overexpressing *TaSnRK2.9* under salt stress. Two-week-old tobacco plants grown on MS medium were transplanted in soil for 3 weeks, plants were watered with 400 mM NaCl solution for 2 weeks. Leaves of the five lines were sampled for the assessment of **(A)** H_2_O_2_, **(B)** SOD, **(C)** CAT, **(D)** POD, **(E)** GSH, **(F)** IL, **(G)** MDA, **(H)** Proline, **(I)** Soluble sugar, **(J)** Chlorophyll. Data are means ± SD (*n* = 3, ^∗^*P* < 0.05; ^∗∗^*P* < 0.01 by Student’s *t*-test).

Under the salt treatment, the SOD, CAT, POD activities, and GSH content in the transgenic plants were remarkably higher than in the control plants, but the H_2_O_2_ content was lower, implying that TaSnRK2.9 could enhance the enzyme activities and antioxidants content to eliminate more ROS (Figures 7A–E). While the IL and MDA contents showed a lower level in the transgenic plants (Figures 7F,G), but the proline, soluble sugar, and the chlorophyll content were significantly higher than the controls (Figures 7H–J). These results indicate that TaSnRK2.9 could help plants produce more osmotic substances to regulate osmosis, to stabilize biopolymers’ structure, and to reduce the cell toxicity, which finally protect plants from seriously salt damage.

### *TaSnRK2.9* Regulates ROS- and ABA-Related Gene Expressions Under Osmotic or NaCl Treatments

**FIGURE 8:**
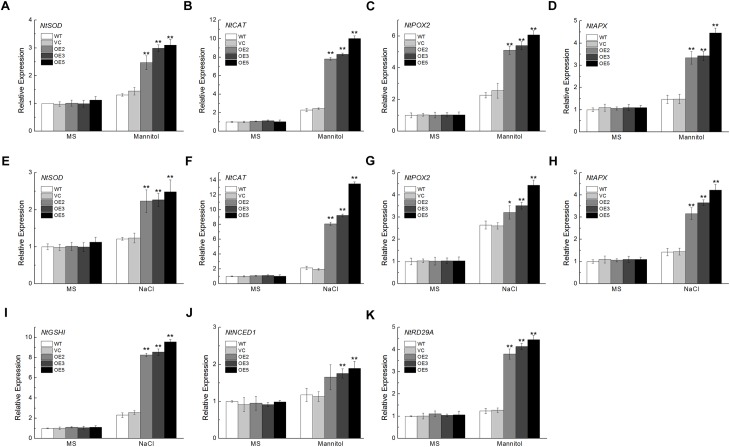
Expression analyses of the ROS-related and ABA-related genes in transgenic tobacco plants overexpressing *TaSnRK2.9* under mannitol or NaCl stress by qRT-PCR. Two-week-old seedlings grown in the MS medium were transplanted to MS medium with 300 mM mannitol or 200 mM NaCl for 1 week. Whole seedlings were sampled for RNA extraction. Expression levels of **(A,E)** *NtSOD*, **(B,F)** *NtCAT*, **(C,G)** *NtPOX2*, **(D,H)** *NtAPX*, **(I)** *NtGSHI*, **(J)** *NtNCED1*, **(K)** *NtRD29A*. Data are means ± SD (*n* = 3, ^∗^*P* < 0.05; ^∗∗^*P* < 0.01 by Student’s *t*-test).

To further investigate the effects of *TaSnRK2.9* in transgenic plants, we examined the expression levels of some ROS- and ABA-related genes in young seedlings with or without stress treatments. ROS-related genes including *NtSOD*, *NtCAT*, *NtPOX2*, *NtAPX*, and *NtGSHI* showed higher levels of expression after mannitol or NaCl treatment in the transgenic plants (Figures 8A–I). Expression of *NtNCED1*, which plays a vital role in ABA biosynthesis, *NtRD29A*, a downstream response gene in the ABA signal pathway, were also increased under mannitol stress (Figures 8J,K). These results demonstrate that TaSnRK2.9 confers tobacco plants abiotic stress tolerance through ROS detoxification, or take part in the ABA signal pathway.

### *TaSnRK2.9* Regulates Stress-Responsive Gene Expressions Under Osmotic or NaCl Treatments

**FIGURE 9:**
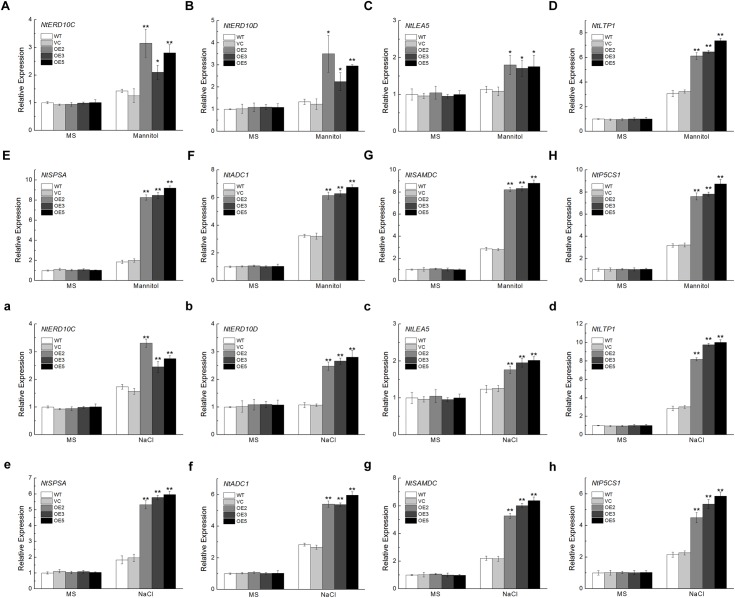
Expression analyses of the stress-responsive genes in transgenic tobacco plants overexpressing *TaSnRK2.9* under normal environment and osmotic stresses. Expression levels of **(A,a)** *NtERD10C*, **(B,b)** *NtERD10D*, **(C,c)** *NtLEA5*, **(D,d)** *NtLTP1*, **(E,e)** *NtSPSA*, **(F,f)** *NtADC1*, **(G,g)** *NtSAMDC*, **(H,h)** *NtP5CS1*. Data are means ± SD (*n* = 3, ^∗^*P* < 0.05; ^∗∗^*P* < 0.01 by Student’s *t*-test).

To get a deeper understanding of the molecular mechanism underlying the improved osmotic or NaCl tolerance in *TaSnRK2.9* overexpression plants, the expression levels of eight stress-responsive genes were detected. These genes include *NtERD10C*, *NtERD10D*, and *NtLEA5* which belong to LEA protein family (Figures 9A–C,a–c), *NtLTP1*, a lipid transfer protein (Figure 9D,d), *NtSPSA*, a sucrose-phosphate synthase (Figure 9E,e), *NtADC1*, a arginine decarboxylase (Figure 9F,f), *NtSAMDC*, a S-adenosyl-L-methionine decarboxylase (Figure 9G,g), *NtP5CS1*, a pyrroline-5-carhoxylate synthase (Figure 9H,h). These selected genes exhibited higher expression levels under osmotic or NaCl treatment than the controls, indicating that TaSnRK2.9 may regulate some stress-response genes to resist drought or salt stress, thus protecting plants from damage.

### TaSnRK2.9 Interacts With NtABF2 and Upregulates the Expression of ABF-Regulated Gene in Transgenic Tobacco Plants

**FIGURE 10:**
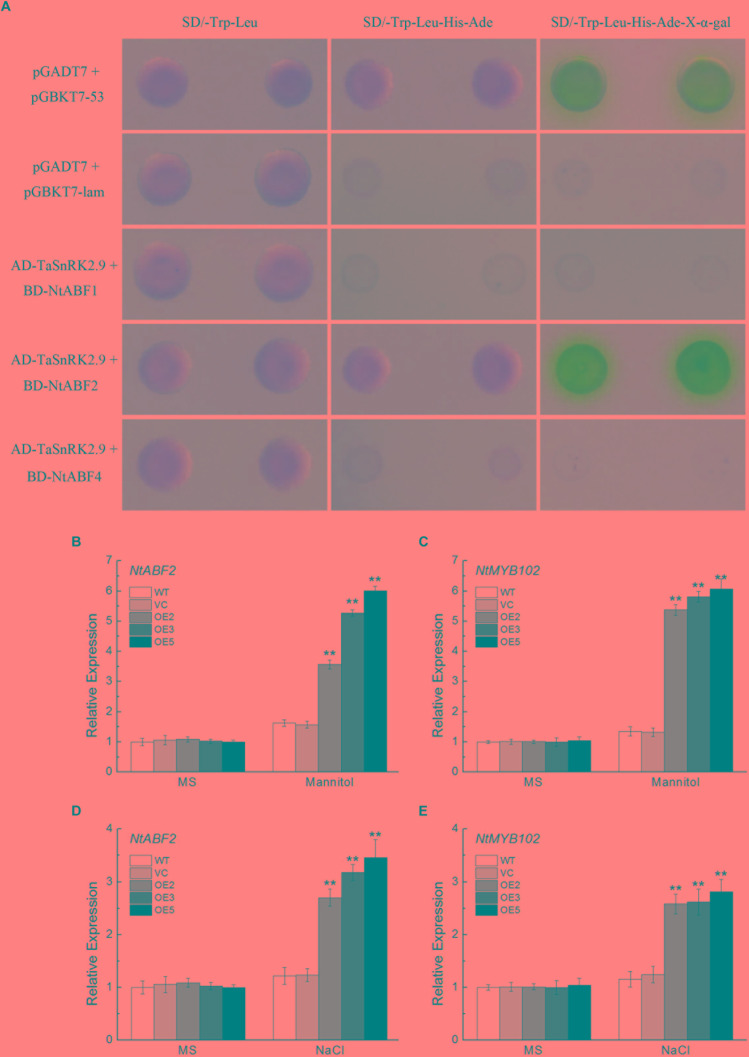
Protein interaction analyses between TaSnRK2.9 and NtABF2 by yeast two-hybrid assay and expression analyses of the related genes. **(A)** *TaSnRK2.9* was cloned into the vector pGADT7, and three *NtABFs* were transferred to vector pGBKT7, then they were co-transformed into the yeast strain AH109. The transformants were grown on nutritional selective medium SD/-Trp-Leu, or SD/-Trp-Leu-His-Ade, or plus the chromogenic substrate X-α-Gal. The first and second lines indicate the positive and negative control, respectively. Three independent biological experiments were performed and produced similar results. **(B,D)** The relative expression level of the predicated interacting gene *NtABF2* under drought or salt stress in tobacco. **(C,E)** The relative expression level of the related gene *NtMYB102*. Data in chart are means ± SD (*n* = 3, ^∗^*P* < 0.05; ^∗∗^*P* < 0.01 by Student’s *t*-test).

To investigate the protein interaction of the TaSnRK2.9 and get a further understanding of the molecular mechanisms in the transgenic plants, yeast two-hybrid assay was used. As shown in Figure 10A, TaSnRK2.9 interacted with NtABF2 in the SD/-Trp-Leu-His-Ade-X-α-gal medium, while TaSnRK2.9 did not interact with NtABF1 or NtABF3. BiFC assay result confirmed this interaction (Supplementary Figure S2). Bright fluorescence was seen through the cell including cytoplasm and nucleus, indicating that TaSnRK 2.9 interacters with NtABF in the tobacco cell. We also tested the expression levels of *NtABF2* and *NtMYB102* (an ABF-regulated gene) under the mannitol or NaCl treatment. Results showed that the expressions of two genes were greatly increased in the *TaSnRK2.9* overexpression plants than the controls (Figures 10B–E). These results suggested that TaSnRK2.9 conferred transgenic tobacco plants tolerance to mannitol or NaCl through interacting with NtABF2 and modulate the ABF-regulated gene expression.

## Discussion

As sessile organisms, plants are exposed to ever changing environments which are harmful to plants growth and development (Zhu, 2016; Vishwakarma et al., 2017). Abiotic stresses such as drought, high salinity, and temperature stresses severely affect cereal crops, and cause the reduction of productivity. The SnRK family members play an indispensable role in plants’ response to stresses. Previous studies of SnRKs mainly focused on ABA-dependent pathway in response to drought and salt stresses (Mao et al., 2010; Zhang et al., 2010, 2011, 2016; Tian et al., 2013). Although some *SnRK* genes in wheat have been identified and studied, the function of *TaSnRK2.9* remains unclear.

In this study, we cloned and characterized a *SnRK2* gene from common wheat, sequences analysis showed that it has a high similarity with *OsSAPK9*, thus designated *TaSnRK2.9*.

### *TaSnRK2.9* Is Responsive to Multiple Stresses and Signal Molecules

Numerous researches have shown that SnRKs are involved in multiple abiotic and biotic stresses. TaSnRKs mainly responded to PEG, NaCl, Cold, and ABA (Mao et al., 2010; Zhang et al., 2010, 2011; Tian et al., 2013). In our work, PEG, NaCl, and H_2_O_2_ rapidly increased the expression of *TaSnRK2.9* (Figures 1A–C), which indicates that *TaSnRK2.9* is sensitive to osmotic stress and reactive oxygen. The signal molecules ABA, MeJA, and ethrel also upregulated the *TaSnRK2.9* expression (Figures 1D–F), suggesting that TaSnRK2.9 may participate in the related signaling pathway.

ABA is an important signal molecule involved in plant abiotic stress. When subjected to abiotic stress, plants produce more ABA, leading to ABA-dependent response (Zhao et al., 2018). Abiotic stress also causes ABA-independent response through *SnRK2s* (Fujii et al., 2011). In *Arabidopsis*, ten SnRK2s family members function redundantly in plant resisting abiotic stress (Fujii et al., 2011). *SnRK2.2*, *SnRK2.3*, *SnRK2.6*, *SnRK2.7*, and *SnRK2.8* are ABA-dependent members (Furihata et al., 2006). MeJA modulates plant growth and defenses against to abiotic stress (Wasternack and Song, 2017). Therefore, understanding how TaSnRK2.9 functions in plant abiotic stress response is important.

### *TaSnRK2.9* Expresses in All Organs in Wheat and the TaSnRK2.9 Protein Locates Throughout the Cell

Unlike other TaSnRKs which mainly expressed in root, organ specific expression analysis shows that *TaSnRK2.9* is mainly expressed in young root, stamen, pistil, and lemma (Figure 1G), indicating that TaSnRK2.9 may play a key role in plant growth and development. Consistent with other TaSnRK2s, TaSnRK2.9 also locates in the whole cell (Figure 2).

### TaSnRK2.9 Enhances Transgenic Tobacco Plants to Drought and Salt Tolerance in Mature Plants and Seedlings

To further investigate the function of TaSnRK2.9 under abiotic stress, we generated transgenic tobacco plants overexpressing *TaSnRK2.9* under the control of *CaMV 35S* promoter. Drought or salt treatment was applied to the young seedlings, mature transgenic plants as well as at seed germination stage. Results showed that *TaSnRK2.9* overexpression tobacco plants exhibited enhanced tolerance to drought or salt treatment in comparison with WT and VC (Figure 3). Similar results were also found during seed germination and at young seedling stage. Previous studies showed the *TaSnRK2.4* transgenic *Arabidopsis* had a delayed seed geimination (Mao et al., 2010). Our research indicated that seeds of *TaSnRK2.9* overexpression tobacco germinated earlier and better than the controls on MS medium with mannitol or NaCl, the final germination rate was higher than the controls (Figure 4), suggesting that TaSnRK2.9 participates in regulating seed dormancy. Overexpression of *TaSnRK2.9* also affected the root length in young tobacco seedlings. Transgenic plants showed longer roots contrast to WT and VC under mannitol or NaCl treatment (Figure 5). This implies that TaSnRK2.9 may help the roots grow longer to get more water and nutrients to tolerate osmotic stress. *TaSnRK2.3*, *TaSnRK2.4*, *TaSnRK2.7*, and *TaSnRK2.8* had the similar effect on the primary root length(Mao et al., 2010; Zhang et al., 2010, 2011; Tian et al., 2013), indicating the SnRK2s have a typical characteristic to promote root elongation.

### The Antioxidant Mechanism and ABA Signal Pathway Are Involved in *TaSnRK2.9* Conferring Osmotic Stress Tolerance

The changes in physiological indices level always indicate the plants’ ability to cope with complex environments. Based on the plants’ phenotype results, physiological indices with or without drought and salt stresses were examined (Figures 6, 7). Abiotic stresses often cause the ROS accumulation, antioxidant enzymes such as SOD, CAT, POD and non-enzymatic antioxidants GSH play crucial roles in ROS scavenging. Plants have evolved a complex system to clear the excessive ROS (Miller et al., 2010). SOD is the first barrier to scavenge ROS by catalyzing the dismutation of O^-2^ to oxygen and H_2_O_2_ with the assistant of CAT and POD (Blokhina et al., 2003). GSH can eliminate free radicals to protect plants from ROS damage and it is a non-enzymatic antioxidant (Wang et al., 2016). Under drought stress, increased RWC indicates that *TaSnRK2.9* overexpression improves plants’ ability to sustain water, helping the cell remain a proper volume and keep a balance to the transpiration rate. Lower IL implies that the cell membrane suffered less damage. MDA, a product of oxidative attack on membrane lipids, is an index of membrane injury, which could change membrane permeability. Proline not only regulates cell osmotic pressure but also modulates cell oxidation-reduction potential, thus safeguards plants from injuring. The chlorophyll content, an important factor of increased biomass and grain yield, increased under salt stress (Figure 7J), indicating the transgenic plants showed higher photosynthetic capacities compared to controls. ABA showed a higher content in the *TaSnRK2.9* overexpression plants under drought stress, suggesting that the accumulated ABA affect the TaSnRK2.9 response to osmotic stress (Figure 6J).

In order to get further insights into how TaSnRK2.9 responds to drought or salt stress at the molecular level, the expressions of five ROS-related and two ABA-related genes were measured (Figure 8). The ROS-scavenging related genes *NtSOD*, *NtCAT*, *NtPOX2*, and *NtAPX* exhibited enhanced expression under mannitol or NaCl condition. The *NtGSHI* was upregulated after NaCl treatment. The expressions of *NtNCED1* and *NtRD29A*, ABA-related genes, were greatly increased under mannitol treatment.

Reactive oxygen species accumulation induced by abiotic stress could damage the plants, so ROS scavenging becomes much more important to maintain the ROS balance (Mittler et al., 2004). Since each subcellular compartment has its own set of ROS production and ROS cleaning pathway, the steady-state level of ROS is not the same at the same time. ROS also acts as signal transduction molecules to modulate diverse pathways when accumulated under stresses (Choudhury et al., 2017). Plants defend abiotic stress *via* a temporal-spatial harmonizing between ROS and other signal molecules such as biological macromolecules, plant hormones (Baxter et al., 2014). When subjected to abiotic stress, the steady-state of the ROS was disturbed, so the plants have evolved a complex mechanism to keep the ROS balance. Overexpression of *TaSnRK2.9* in tobacco plants could scavenge ROS *via* antioxidant enzymes (SOD, CAT, POD) and non-enzymatic antioxidant (GSH), thus sustaining ROS to a normal level to defend abiotic stress.

Abscisic acid signal pathway is widely studied, and plants defense against abiotic stress *via* ABA-dependent or ABA-independent pathway. *SnRK2.2*, *SnRK2.3*, and *SnRK2.6* regulate the plant tolerance to abiotic stress through ABA-dependent way, but whether *TaSnRK2.9* works in the same way remains unknown. In our present research, *TaSnRK2.9* can be induced by ABA, overexpression of *TaSnRK2.9* caused ABA accumulation, and elevated the expressions of ABA-related genes. These results indicate TaSnRK2.9 functions through ABA-dependent pathway when subjected to abiotic stress.

### *TaSnRK2.9* Regulates the Expressions of Some Stress-Response Genes in Transgenic Tobacco

To better understand the function of TaSnRK2.9 against abiotic stress, we examined the expressions of some stress-response genes. Eight such genes examined showed significantly higher expression than the controls under stress conditions. *NtERD10C*, *NtERD10D*, and *NtLEA5*, belonging to LEA family, could protect the structure of cell membranes and stabilize the biomacromolecules (Xiong and Zhu, 2002; Amara et al., 2012; Liu et al., 2013). The LEA protein family comprises a lot of multifunction proteins which is considered necessary in plants response to abiotic stress (Mertens et al., 2018). *NtLTP1* encodes a lip transfer protein, which is involved in plant defending abiotic stress (Hu et al., 2013). As the cell membranes are mainly made up of lipid bilayer, the improved *NtLTP1* expression indicates that plants produces more lip transfer protein, thus protect the cell membranes from severely damage. *NtSPSA* plays an important role in the biosynthesis of sucrose (Lin et al., 2008). The increased sucrose could provide more nutrients to plants when subjected to abiotic stress and supported plants growth and development (Chen et al., 2005) and could maintain osomatic balance. *NtADC1* and *NtSAMDC* participate in the biosynthesis of polyamine, which could regulate cell osmotic pressure and stabilize the membrane structure (Jang et al., 2009). *NtP5CS1* is a key regulator in proline biosynthesis (Dorothea and Ramanjulu, 2005). The expressions of stress-response genes were increased under mannitol or NaCl treatment in the *TaSnRK2.9* overexpression plants, while the plants grown in the MS medium showed no difference (Figure 9). *TaSnRK2.9* transgenic plants exhibited enhanced stress tolerance and produced more stress-response proteins when exposed to abiotic stress, which suggests that these proteins and sugar play essential roles in mediating plants against abiotic stress. In a word, *TaSnRK2.9* regulates the expression of multiple stress-response genes and guarantees plants better adaption to the stress environment.

### TaSnRK2.9 Is Involved in SnRK-ABF Pathway to Enhance Tolerance to Drought and Salt Stresses in Transgenic Tobacco Plants

To investigate the mechanism of *TaSnRK2.9* action, we also conducted yeast two-hybrid assay. In the previous studies, interactions between AtSnRKs (SnRK2.2, SnRK2.3, SnRK2.6) and AtAREB1/ABF2 were identified, meanwhile many AtABFs and AtSnRK2s can be simultaneously regulated both by ABA and abiotic stresses (Kobayashi et al., 2010; Chan, 2012). Accumulated ABA modulates the expression of many genes controlled by ABA-responsive elements (ABREs) when subjected to abiotic stress. ABRE-binding protein/ABRE-binding factor (AREB/ABF) plays a key role in pants response to tolerance *via* regulating a series of gene expressions (Fujita et al., 2013). The SnRKs (SnRK2.2, SnRK2.3, SnRK2.6) protein could actively regulate the AREB/ABF transcription factors (Furihata et al., 2006; Fujii et al., 2007; Fujii and Zhu, 2009). AREB1, AREB2, and ABF3 are the master transcription factors that cooperatively regulate ABRE-dependent gene expression for ABA signaling under drought stress (Yoshida et al., 2010). So the SnRK-ABF pathway has a great importance in plants resisting abiotic stress (Wang L. et al., 2015). In our research, TaSnRK2.9 could interact with NtABF2, and upregulated the expression of *NtABF2* under mannitol or NaCl treatment (Figures 10A,B,D). MYB102, a transcription factor containing a conserved MYB DNA-binding domain, which could be regulated by ABF, is involved in plants stress response (Stracke et al., 2001; Yoshida et al., 2010). The ABF-related gene *NtMYB102* showed a great increase after mannitol or NaCl treatment (Figures 10C,E).

In the present study, we found that ABA could induce the expression of *TaSnRK2.9* (Figure 1D), suggesting that the function of TaSnRK2.9 is connected with ABA. When plants were subjected to drought stress, *TaSnRK2.9* overexpression caused more ABA accumulation (Figure 6J), and induced the expressions of ABA-related genes (Figures 8J,K), while the accumulated ABA caused a lot of downstream gene expressions and other physiological response so that to adjust to the abiotic stress response. These results indicate that *TaSnRK2.9* may participate in the plants stress response *via* an ABA-dependent pathway. Furthermore, our result showed that TaSnRK2.9 interacted with NtABF2, and *TaSnRK2.9* overexpression can upregulate the expressions of *NtABF2* and ABF-related gene *NtMYB102* under mannitol or NaCl (Figure 10). These implies that TaSnRK2.9 specifically interact with NtABF2 in response to ABA and osmotic stress.

Overexpression of *TaSnRK2.9* enhanced tobacco plants tolerance to multiple abiotic stresses, thus making *TaSnRK2.9* a candidate gene for molecular breeding and it is promising to improve crop plant stress resistance by using genetic engineering.

## Conclusion

TaSnRK2.9 was responsive to various abiotic stress and different signaling molecules. Overexpression of *TaSnRK2.9* enhanced tobacco plants tolerance to drought or salt with improved antioxidant system, RWC, proline, soluble sugar, chlorophyll, and ABA content, reduced H_2_O_2_, IL, and MDA. In summary, TaSnRK2.9 could enhance ROS scavenging ability through ABA-dependent signal transduction and specific SnRK-ABF interaction, resulting in enhanced plants tolerance to abiotic stress in transgenic tobacco plants.

## Author Contributions

JF and LW designed all the experiments. JF analyzed the data and wrote the manuscript. LW conducted the research technology. YW carried out the stress tolerance experiments. QL and YZ help with making figures. DQ and JH participated in the bioinformatics analyses. PS, ZX, and JC conducted the PCR. GY and GH conceived the study and revised the manuscript. All the authors read and approved the final manuscript.

## Conflict of Interest Statement

The authors declare that the research was conducted in the absence of any commercial or financial relationships that could be construed as a potential conflict of interest.

## Footnotes

1 https://www.ncbi.nlm.nih.gov/

2 http://plants.ensembl.org/index.html

## Abbreviations

ABA: abscisic acid
ABF: ABA-responsive element-binding transcription factor
AMPK: AMP-activated protein kinase
CaMK: calmodulin-dependent protein kinases
CaMV: cauliflower mosaic virus
CAT: catalase
CCaMK: calcium and calmodulin-dependent protein kinases
CDPK: calcium-dependent protein kinases
CRK: CDPK-related kinases
EST: expressed sequence tag
GFP: green fluorescent protein
GSH: glutathione
IL: ion leakage
LEA: late embryogenesis-abundant
MDA: malondialdehyde
MeJA: methyl jasmonate
MS: Murashige and Skoog
OE: overexpression
ORF: open reading frame
PEG: polyethylene glycol
PEPRK: PEP carboxylase kinase-related kinases
POD: peroxidase
PP2C: type 2C phosphatase
PPCK: phosphoenolpyruvate carboxylase kinases
PYL: PYR-like
PYR: pyrabactin resistance
qRT-PCR: quantitative reverse-transcription PCR
RACE: rapid amplification of cDNA ends
RCAR: regulatory component of ABA receptor
ROS: reactive oxygen species
RWC: relative water content
SNF: sucrose non-fermenting
SnRK: sucrose non-fermenting 1-related protein kinase
SOD: superoxide dismutase
VC: vector control
WT: wild type
