# Supplementary material for: TaSnRK2.9, a Sucrose Non-fermenting 1-Related Protein Kinase Gene, Positively Regulates Plant Response to Drought and Salt Stress in Transgenic Tobacco
Source: Front Plant Sci. 2019 Jan 14;9:2003. doi: 10.3389/fpls.2018.02003 (PMC6339923; doi:10.3389/fpls.2018.02003)
Supplement: Supplementary file 1 [file Table_1.DOC]

Table S1. Primers used in this study

| **Name** | **Sequence (5' to 3')** | **Description and purpose** |
| --- | --- | --- |
| *TaSnRK2.9* | GGGATCTCCGTGTCTTGGTC | Forward primer for full-length |
|  | CGGCACTCAATCTGGTTCGT | Reverse primer for full-length |
|  | TGGATGACCTGGACTCGG | Forward primer for real time qRT-PCR |
|  | AAGCAAACAAGGTGGAAGAC | Reverse primer for real time qRT -PCR |
|  | GAAGATCTATGGAGAGGGGGCCGG | Forward primer for cloning gene in pCAMBIA1304 |
|  | CGGACTAGTACATGGCGTATACTATC | Reverse primer for cloning gene in pCAMBIA1304 |
|  | GGAATTCCATATGATGGAGAGGGGGCCGG | Forward primer for cloning gene in pGADT7 |
|  | CCGGAATTCCTACATGGCGTATACTA | Reverse primer for cloning gene in pGADT7 |
| *NtABF1* | CGGAATTCATGGGATCTCAGGGTGGTGG | Forward primer for cloning gene in pGBKT7 |
|  | CGGGATCCTTAGAAAGGGGCGGAGCTTG | Reverse primer for cloning gene in pGBKT7 |
| *NtABF2* | CGGAATTCATGGGGAGTAATTTTAATTTC | Forward primer for cloning gene in pGBKT7 |
|  | CGGGATCCTTACCATGGACCAGTCTGTG | Reverse primer for cloning gene in pGBKT7 |
| *NtABF4* | CGGAATTCATGCAATATTGTTTACTGGTG | Forward primer for cloning gene in pGBKT7 |
|  | ACGCGTCGACCTACCAAGGTCCTGTCACTG | Reverse primer for cloning gene in pGBKT7 |
| *TaACTIN* | AGTGGAGGTTCTACCATGTTTCCT | Forward primer for real time qRT-PCR |
|  | CACTGTATTTCCTTTCAGGTGGTG | Reverse primer for real time qRT -PCR |
| *NtACTIN* | CTATTCTCCGCTTTGGACTTGGCA | Forward primer for real time qRT-PCR |
|  | ACCTGCTGGAAGGTGCTGAGGGAA | Reverse primer for real time qRT -PCR |
| *NtSOD* | CTCCTACCGTCGCCAAAT | Forward primer for real time qRT-PCR |
|  | GCCCAACCAAGAGAACCC | Reverse primer for real time qRT -PCR |
| *NtCAT* | AGGTACCGCTCATTCACACC | Forward primer for real time qRT-PCR |
|  | AAGCAAGCTTTTGACCCAGA | Reverse primer for real time qRT -PCR |
| *NtPOX2* | ATAGGAACACTTATGAAG | Forward primer for real time qRT-PCR |
|  | GAGATAATGGTTGAGTTA | Reverse primer for real time qRT -PCR |
| *NtAPX* | GACATTGCTATCAGACTC | Forward primer for real time qRT-PCR |
|  | CTCCAGTAACTTCAACAG | Reverse primer for real time qRT -PCR |
| *NtGSHI* | GAGTGCATTTCAATCCAGT | Forward primer for real time qRT-PCR |
|  | ATTTATTTCATTTTGCATGGCTA | Reverse primer for real time qRT -PCR |
| *NtNCED1* | AAGAATGGCTCCGCAAGTTA | Forward primer for real time qRT-PCR |
|  | GCCTAGCAATTCCAGAGTGG | Reverse primer for real time qRT -PCR |
| *NtRD29A* | TCGGTGTACCAACAGGCATA | Forward primer for real time qRT-PCR |
|  | CCCTTGCTTTGGTGTTGTTT | Reverse primer for real time qRT -PCR |
| *NtERD10C* | AACGTGGAGGCTACAGATCG | Forward primer for real time qRT-PCR |
|  | GTTCCTCTTGGGCATGAGTT | Reverse primer for real time qRT -PCR |
| *NtERD10D* | GAGGACACGGCTGTACCAGT | Forward primer for real time qRT-PCR |
|  | GCGCCACTTCCTCTGTCTT | Reverse primer for real time qRT -PCR |
| *NtLEA5* | TTGAATCTGGGGTTTTGGTT | Forward primer for real time qRT-PCR |
|  | GGAAGCATTGACGAGCTAGG | Reverse primer for real time qRT -PCR |
| *NtLTP1* | GCAGAAGCCATAACCTGTGG | Forward primer for real time qRT-PCR |
|  | CAGTGGAAGGGCTGATCTTG | Reverse primer for real time qRT -PCR |
| *NtSPSA* | GAATTCAGGCGCTTCGTTGTCA | Forward primer for real time qRT-PCR |
|  | ACCCCTAGTTTCTCCAGTGA | Reverse primer for real time qRT -PCR |
| *NtADC1* | CTTGCTGATTACCGCAATTTATC | Forward primer for real time qRT-PCR |
|  | TAGGATCAGCAGCCCCCATAGCC | Reverse primer for real time qRT -PCR |
| *NtSAMDC* | CATTCACATTACCCCGGAAG | Forward primer for real time qRT-PCR |
|  | AGCAACATCAGCATGCAAAG | Reverse primer for real time qRT -PCR |
| *NtP5CS1* | ATCTTCTAGTTCTGTTGA | Forward primer for real time qRT-PCR |
|  | CTCTCCTTAATGTATGTG | Reverse primer for real time qRT -PCR |
| *NtABF2* | CCTAAGTATCAGCTGCGTA | Forward primer for real time qRT-PCR |
|  | AATTTCAATGACATAACGAAC | Reverse primer for real time qRT -PCR |
| *NtMYB102* | CTCAACTTACTATAACAACAGC | Forward primer for real time qRT-PCR |
|  | TTCCGAACTGTATTATCTGGT | Reverse primer for real time qRT –PCR |
|  |  |  |
