# Supplementary material for: TaSnRK2.9, a Sucrose Non-fermenting 1-Related Protein Kinase Gene, Positively Regulates Plant Response to Drought and Salt Stress in Transgenic Tobacco
Source: Front Plant Sci. 2019 Jan 14;9:2003. doi: 10.3389/fpls.2018.02003 (PMC6339923; doi:10.3389/fpls.2018.02003)
Supplement: Supplementary file 2 [file Data_Sheet_1.PDF]

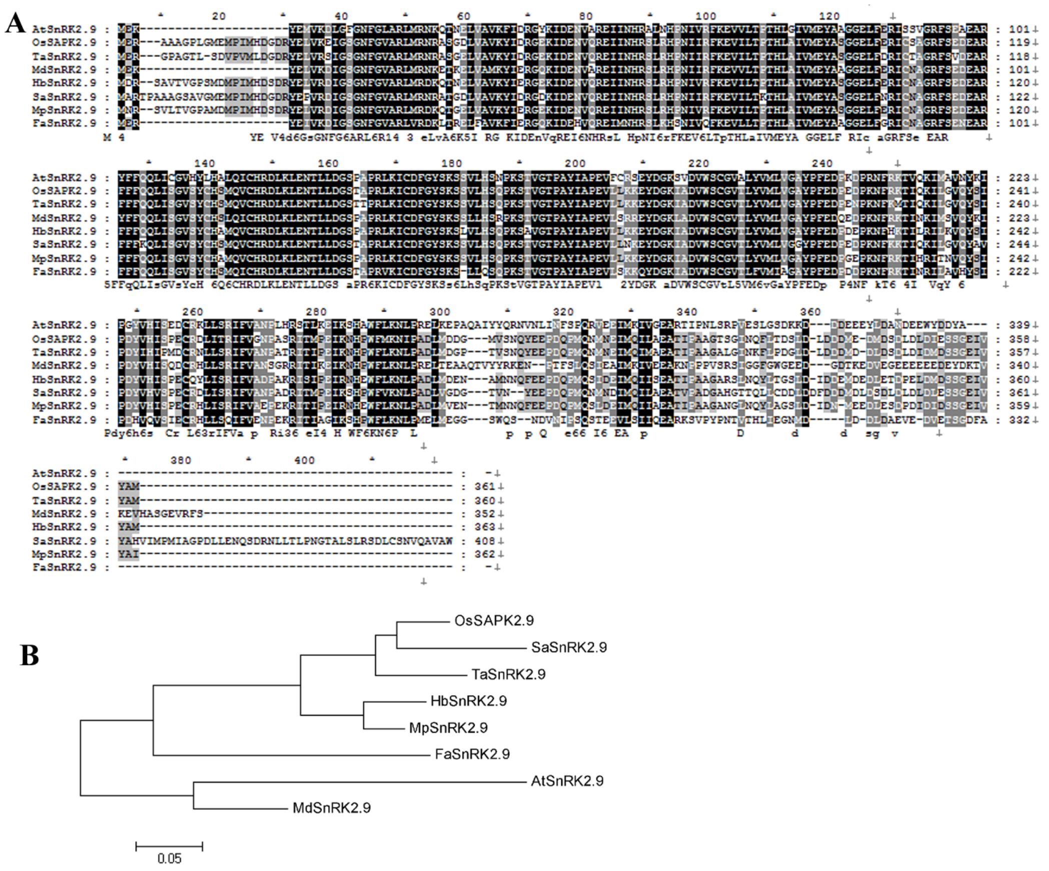

Figure S1. Phylogenetic tree, sequence alignment of SnRK2.9s in different plant species. (A) SnRK2.9 amino acid sequence from *A. thaliana*, *O. sativa*, *T. aestivum*, *Malus domestica*, *Hevea brasiliensis*, *Saccharum* hybrid cultivar, *Malus prunifolia* and *Fragaria* x ananassa. (B) Phylogenetic tree of SnRK2.9s.
