# Supplementary material for: TaSnRK2.9, a Sucrose Non-fermenting 1-Related Protein Kinase Gene, Positively Regulates Plant Response to Drought and Salt Stress in Transgenic Tobacco
Source: Front Plant Sci. 2019 Jan 14;9:2003. doi: 10.3389/fpls.2018.02003 (PMC6339923; doi:10.3389/fpls.2018.02003)
Supplement: Supplementary file 3 [file Data_Sheet_2.PDF]

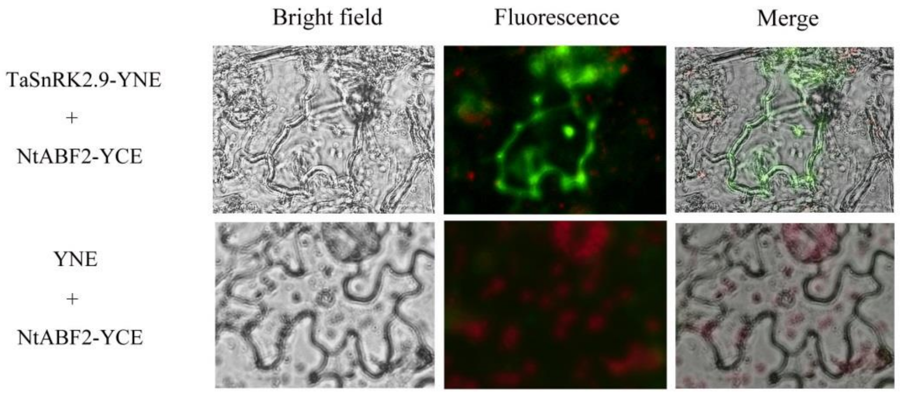

Figure S2. **BIFC of TaSnRK2.9 interaction with NtABF2 transiently expresses in tobacco leaves.** YNE and NtABF2-YCE was set as negative control. Red fluorescent signal is from chlorophyll autofluorescence.
